# Loop Interrupted: Dysfunctional Chromatin Relations in Neurological Diseases

**DOI:** 10.3389/fgene.2021.732033

**Published:** 2021-08-05

**Authors:** Marthe Behrends, Olivia Engmann

**Affiliations:** ^1^Faculty of Medicine, Friedrich Schiller Universität, Jena, Thüringen, Germany; ^2^Jena University Hospital, Institute for Human Genetics, Thüringen, Germany

**Keywords:** chromatin architecture, looping, mental illness, addiction, Alzheimer’s disease

## Abstract

The majority of genetic variants for psychiatric disorders have been found within non-coding genomic regions. Physical interactions of gene promoters with distant regulatory elements carrying risk alleles may explain how the latter affect gene expression. Recently, whole genome maps of long-range chromosomal contacts from human postmortem brains have been integrated with gene sequence and chromatin accessibility data to decipher disease-specific alterations in chromatin architecture. Cell culture and rodent models provide a causal link between chromatin conformation, long-range chromosomal contacts, gene expression, and disease phenotype. Here, we give an overview of the techniques used to study chromatin contacts and their limitations in brain research. We present evidence for three-dimensional genome changes in physiological brain function and assess how its disturbance contributes to psychiatric disorders. Lastly, we discuss remaining questions and future research directions with a focus on clinical applications.

## The 3D Genome

The linear sequence of DNA contains the information to build individual components of a cell, tissue or organism. However, the processes of cell differentiation, tissue formation and life itself are governed by the selective use of this information. After all, species complexity correlates with the number of gene regulatory mechanisms and the prevalence of non-coding sequences rather than gene number (Miele and Dekker, 2008). Thus, in order to understand aberrant protein expression, one must consider the gene as a subject of its nuclear environment. This is crucial for the gain of cellular identity in every tissue, however, as the brain is dynamic, the nature of its epigenetic modifications – including the 3D chromatin architecture – are of special interest as they hold the key to the etiology of neurologic and psychiatric disorders.

The cell nucleus is a highly ordered structure that contributes to cellular identity through cell type-specific spatial organization of chromatin (Dekker and Mirny, 2016). Chromosomes occupy defined spaces – “chromosome territories” – that determine their interaction with the nuclear lamina and each other (Watson and Tsai, 2017). Interaction with the nuclear lamina tends to have a silencing effect on gene expression (Watson and Tsai, 2017; Espeso-Gil et al., 2020), whereas interchromosomal contacts may contribute to either repression or activation (Miele and Dekker, 2008). The latter does not occur through general chromosome amalgamation but is specific to small regions that extrude from the chromosomes in order to come into physical proximity with DNA sequences on other chromosomes (Dekker, 2003). These trans-interactions are commonly observed on smaller chromosomes and occur much less frequently than intrachromosomal contacts (Mitchell et al., 2016). Intrachromosomal contacts include ‘chromatin loops’, which connect DNA regions that are separated on the linear genome on the scale of kilo- or, for some loci, even megabases (Dekker, 2003). However, the majority of intrachromosomal loops is comprised of interaction between two relatively short – typically 5-10kb – distant sequences that stand out from their linear genomic neighborhood (Rajarajan et al., 2018a). Their primary function appears to be the juxtaposition of gene regulatory elements with the promoters of their target genes to control transcriptional activity (Dekker, 2003). This allows enhancer sequences rich in protein binding motifs to place their binding factors into spatial proximity with the promoter, which may lead to activation, if the enhancer has loaded transcription factors (TFs), or repression (Rajarajan et al., 2018b). Generally, activating histone marks around interacting sequences tend to promote looping (Bharadwaj et al., 2014; Engmann et al., 2017; Mitchell et al., 2018).

Enhancers do not only serve as scaffolds for protein binding – many of them undergo active transcription to produce enhancer RNAs (eRNAs) (Sartorelli and Lauberth, 2020). eRNAs have been shown to act on distant loci, but more commonly alter the activity of their origin enhancer or adjacent promoters (Sartorelli and Lauberth, 2020). It is a matter of current debate whether the eRNAs or the act of enhancer transcription itself are required for a functional outcome at the parent enhancer (Sartorelli and Lauberth, 2020). Preliminary research indicates that eRNAs can interact with cohesin and mediator complexes to directly influence chromatin looping (Sartorelli and Lauberth, 2020). Furthermore, eRNAs can promote the activity of cAMP response element-binding protein (CBP), which is a histone acetyltransferase (Sartorelli and Lauberth, 2020). Thus, eRNAs are suspected to influence loop formation by promoting a more open chromatin state and through cohesin stabilization. eRNAs are a rather novel player in chromatin remodeling and their mechanisms of action are not yet completely deciphered. Hence, we will not engage in further discussion about eRNAs but ask the readers to keep in mind that chromatin status around an enhancer will influence looping and/or enhancer transcription. Similarly, SNPs in enhancer sequences may prevent TF binding, loop formation and/or eRNA function.

Within the cell nucleus, many regulatory sequences may compete for interaction with, or act synergistically on a given promoter. Thus, loop formation is likely the result of many integrated signals that together orchestrate gene expression depending on the state of the cell (Miele and Dekker, 2008; Rajarajan et al., 2018b). Considering the complexity of the nuclear interior it would be unlikely for a promoter-enhancer pair to encounter each other by random collision. Indeed, Dekker and Mirny suggest that the relative inflexibility of chromatin contributes to the spatial isolation of loci and thus, prevents an aberrant formation of chromosomal contacts (Dekker and Mirny, 2016).

Loops are stabilized by CCCTC-binding factor (CTCF), a zinc finger protein that is known for its pivotal role in chromatin architecture (Rajarajan et al., 2018b). In the context of chromatin looping, CTCF serves as a scaffold for the attachment of cohesin proteins that form a ring structure around the DNA strands, which has been associated with cell-type specific gene expression and formation of higher order chromatin structures (Mitchell et al., 2014; Rajarajan et al., 2018b). Specifically, the formation and maintenance of topologically associating domains (TADs) depends on CTCF/cohesin (Rajarajan et al., 2019). Topologically associating domains are self-assembling chromatin terrains spanning 100s of kilobases that have high chromosomal contact frequencies within them compared to contacts with the rest of the genome and thus help to prevent aberrant chromatin interactions (Dekker and Mirny, 2016). Interestingly, overall TAD structure appears to be fairly invariant across cell- or tissue types, yet the inter-/intrachromosomal contacts within them change during differentiation (Dekker and Mirny, 2016; Rajarajan et al., 2019). This suggests that TAD assembly has a more static role for nuclear organization. Additionally, histone modifications tend to be similar across a TAD and thus the degree of chromatin compaction is a TAD-associated property (Watson and Tsai, 2017). This is the foundation for the large eu-/heterochromatic compartments that are formed through clustering of multiple TADs (Miele and Dekker, 2008; Dekker and Mirny, 2016; Rajarajan et al., 2019). Therefore, transcriptional activity is restricted to specialized compartments – “active neighborhoods” or “transcriptional hubs” – that share TFs and other factors needed for transcription and allow the coordinated expression of specific gene groups (Miele and Dekker, 2008). Similarly, the clustering of telomeric heterochromatin correlates with high concentrations of silencing factors to achieve co-repression (Miele and Dekker, 2008). The respective epigenetic landscape around a gene locus determines not only its accessibility for TFs and RNA polymerase 2, but also its availability for distant interaction partners (Mitchell et al., 2014).

## Illuminating the Loop

Much of our current knowledge about long range DNA interactions has been generated through the use of chromatin conformation capture (3C) and techniques derived from it. 3C measures the average contact frequency between two DNA sequences within a cell population (Dekker, 2003). The chromatin conformation at the time of testing is preserved by crosslinking proteins and DNA with formaldehyde, followed by a restriction digest of the DNA and re-ligation under dilute conditions (Dekker, 2003). The latter step is crucial as it preferentially ligates cross-linked restriction fragments – the DNA elements that were in close spatial proximity when the nuclei were fixed (Dekker, 2003). Lastly, quantitative polymerase chain reaction (PCR) measures the amount of a ligation product, which is proportional to the interaction frequency between the two target loci (Dekker, 2003). This labor-intensive technique is useful to prove the interaction of candidate loci as their sequences must be known for primer design (Dekker, 2003; van Berkum and Dekker, 2009). The same is true for circularized chromosome conformation capture (4C), where reverse PCR on circularized ligation fragments and next generation sequencing are combined to elucidate all long range interactions involving a candidate locus (Krijger et al., 2020). 3C carbon copy (5C) overcomes restraints imposed by locus selection through the concurrent use of thousands of primers, whose PCR products are then analyzed using deep sequencing (van Berkum and Dekker, 2009). 5C can be used to measure interactions of “many to many” loci but still requires the choice of specific primers, commonly corresponding to fragments of a larger genomic region of interest and thus allowing the investigation of regulatory networks within that genomic region (van Berkum and Dekker, 2009). To explore long-range interactions across the entire genome, HiC is employed. In this 3C derivative, restriction fragments are labeled with biotin prior to ligation and an enzyme is used to remove the biotin post-ligation, allowing for enrichment of the properly ligated fragments before sequencing (Kempfer and Pombo, 2020). The toolbox exists, so why do we know so little about chromatin looping in the brain? An inherent problem with studying DNA – be it chromosomal contacts, or the proteome associated with specific genomic loci – is the limited amount of material (Gauchier et al., 2020). A genomic locus will be present twice in a diploid cell, whereas there are potentially hundreds or even thousands of mRNA molecules or proteins present (Gauchier et al., 2020). Therefore, 3C based approaches require large cell numbers (∼3 million for HiC) as input (Kempfer and Pombo, 2020), which will take several weeks to grow in neuronal culture (Mitchell et al., 2014). Rodent models are a valuable tool to study long range chromosomal contacts because they allow for casual interference using transgenes and pharmaceutical agents at a minimal postmortem delay. However, in order to harvest enough neurons, animal studies may require pooling of tissue, which reduces the power of the data and raises ethical concerns regarding the number of sacrificed animals. Human postmortem brains can more easily provide high cell numbers, and results are directly relevant to patient cohorts. However, samples can be hard to obtain, are often heterogeneous and have long postmortem delays.

The *in vivo* study of brain loops is further complicated through cell heterogeneity, which can be circumvented by cell sorting approaches (Mitchell et al., 2014). Most importantly, isolated nuclei can be subjected to fluorescence-activated nuclei sorting (FANS). This technique utilizes fluorescence labeled antibodies against the neuron-specific splicing factor NeuN, which allows for high purity samples of neuronal chromatin, even from postmortem tissue (Dammer et al., 2013). This has facilitated the investigation of long-range chromosomal interactions within comparatively large brain compartments such as the hippocampus and prefrontal cortex. Recently, HiC protocols have been adapted for low cell numbers (∼300,000 cells) (Kempfer and Pombo, 2020) and for the first time chromosomal contacts in a rare neuronal population, the midbrain dopaminergic neurons, were successfully analyzed (Espeso-Gil et al., 2020). Studying chromosomal interactions within brain nuclei remains a time- and cost intensive endeavor. In particular, the price for deep sequencing of genome-wide contacts prevents many laboratories from adding 3D studies of the genome to their methodological repertoire. Nevertheless, investigation of global chromatin contacts is made feasible by the active cooperation of many research groups that compile and integrate their results in publicly available databases. Among them is the Psychencode project that holds information on the transcriptome and epigenetic landscape of ∼2000 human postmortem brains derived from patients with various psychiatric disorders (Rajarajan et al., 2019). Recently, HiC data derived from fetal and adult brain have been made available (Rajarajan et al., 2019). This pioneering project is one of many invaluable resources that allow the in-depth study of the human (epi-)genome in the context of brain function and disease (see Box 1).

**Box 1. Publicly Available Datasets on chromatin architecture.**

**Encyclopedia of DNA Elements (ENCODE)** https://www.ct.org/data/annotations/

ENCODE comprises an annotation of the human genome across cell types with respect to chromatin accessibility, histone marks, TF binding, mRNA expression, DNA methylation, 3D chromatin interactions and TADs. These data were used to identify *cis* regulatory elements active in specific cell types and to assign chromatin states to genomic regions.

**Roadmap Epigenomics Project** http://www.roadmapepigenomics.org/

The project is aimed to map the reference epigenome of adult and fetal human cell types. It currently contains information on DNA methylation, histone marks and overall chromatin accessibility in various fetal and adult brain regions and major human organs under physiological conditions.

**Brain Cloud**

https://www.ncbi.nlm.nih.gov/projects/gap/cgi-bin/study.cgi?study_id=phs000417.v2.p1

The mRNA expression and DNA methylation data are derived from the dorsolateral prefrontal cortex of ∼270 individuals ranging from 8 weeks to 80 years of age.

**Psychiatric Genomics Consortium** https://www.med.unc.edu/pgc/

This data base encompasses large scale GWAS studies focused on a wide number of psychiatric diseases: ADHD, Alzheimer’s disease, autism, bipolar disorder, eating disorders, major depressive disorder, obsessive-compulsive disorder/Tourette syndrome, post-traumatic stress disorder, schizophrenia, substance use disorders, and anxiety disorders. The global network of scientists has identified disease-associated risk loci and provides primary data upon request.

**Psychencode Project** http://www.psychencode.org/?page_id=21

DNA sequences, transcriptomic and epigenetic data from 2000 postmortem adult and developing brains are integrated. Samples include healthy controls, SCZ patients, individuals suffering from autism spectrum disorder or subjects with bipolar disorder.

**Common Mind Consortium** https://www.synapse.org/#!Synapse:syn2759792/wiki/197295

RNAseq, DNAseq, ATACseq, genotyping, epigenetic data generated from different regions of ∼1000 postmortem brains were derived from patients suffering from SCZ and bipolar disorder and neurotypical controls.

**Greek LOGOS (Learning on Genetics of Schizophrenia Spectrum) Project** https://www.sciencedirect.com/science/article/pii/S0006322310009443

https://www.nature.com/articles/npp201149

445 healthy young males (Greek army conscripts) were selected based on a psychological questionnaire. Their genotype was assessed with respect to six SCZ-related SNPs and pre-pulse inhibition was measured.

**SZDB** http://www.szdb.org/

Data were compiled from numerous studies focused on SCZ. Integrated genetic, gene expression, brain eQTL, SNP function data to identify genes and pathways contributing to SCZ. The recent version also contains a polygenic risk score calculator.

## Worth Every Penny

Only a dynamic epigenetic landscape – in contrast to the static DNA sequence – can provide the foundation for neuronal plasticity (Watson and Tsai, 2017). The characterization of chromatin architecture is therefore of fundamental interest. Chromatin alterations can occur as a response to neuronal activity (Watson and Tsai, 2017) and differentiation (Rajarajan et al., 2018a). Thus, chromatin loop remodeling is expected to occur in brain development and disease as well. Rajarajan and colleagues used human induced pluripotent stem cells (hIPSCs) that were differentiated into neuronal progenitor cells (NPCs) and finally neurons or glia as an *in vitro* model of brain development (Rajarajan et al., 2018a). HiC analysis of these cells revealed that lineage commitment correlates with large scale remodeling of long-range chromosomal contacts (Rajarajan et al., 2018a). Neurons specifically underwent substantial “pruning” of chromatin loops to adapt a cell-type-specific transcriptome (Rajarajan et al., 2018a). Work from the same group suggests that non-random nuclear compartmentalization is relevant to connect specific cellular processes in neurons: mapping of the 3D-genome of midbrain dopaminergic neurons demonstrated a co-localization of genes implicated in cognition and lipid metabolism to specific chromatin domains (Espeso-Gil et al., 2020).

The relevance of looping for basic neuronal function is further underscored by its influence on N-methyl-D-aspartate receptor (NMDAR) expression. NMDARs are crucial for normal synaptic transmission and facilitate learning and memory (Zhu et al., 2016). Expression of the Glutamate [NMDA] receptor subunit (Grin2b) has been shown to depend on long-range loops specific to the prefrontal cortex (PFC) (Bharadwaj et al., 2014). The Grin2b transcription start site (TSS) interacts with a proximal (∼55kb distance) intergenic sequence that represses Grin2b in most tissues (Bharadwaj et al., 2014). However, in PFC neurons, two distant enhancers compete with the proximal repressor for Grin2b TSS binding (Bharadwaj et al., 2014). The enhancers are loaded with transcriptional activators – namely activating protein 1 (AP1) and nuclear respiratory factor 1 (NRF1) – that induce a brain-specific transcription of Grin2b (Bharadwaj et al., 2014).

The work discussed above describes long-term transcriptional effects brought about by altered chromatin loops in the context of brain development. However, the role of long-range chromosomal interactions to facilitate temporary changes in gene expression and thus quotidian cell function is even less understood. A ground-breaking finding was made by Sassone-Corsi’s group, who traced long-range chromosomal contacts across the course of a day in an *in vitro* system (Aguilar-Arnal et al., 2013). Their work suggests that genes, whose expression is controlled by the central clock, are part of a stably interacting looping network with rhythmically oscillating contact frequencies that correlate with circadian transcriptomic changes (Aguilar-Arnal et al., 2013). The authors showed that the cadent nature of the interactome depends on Brain and Muscle ARNT-Like 1 (BMAL1) and thus concluded that the “clock machinery itself” may form the basis for circadian loops and hence gene expression changes (Aguilar-Arnal et al., 2013). Recent research increasingly implicates the 3D genome as a regulator of circadian transcription activity in peripheral tissues, but data on the central pacemaker is lacking (Pacheco-Bernal et al., 2019). Nevertheless, it can be inferred that chromatin looping contributes to the temporal harmonization of fundamental brain functions.

## Specific Chromatin Interactomes in the Etiology of Neurologic Dysfunction

Genome-wide association studies (GWAS) have identified genetic variants that are associated with neurologic disorders such as Schizophrenia (SCZ), Alzheimer’s (AD) and many more. Single nucleotide polymorphisms (SNPs) commonly lie outside of gene sequences but may interact with expressed gene targets via chromatin loops to change their expression (Rajarajan et al., 2018b). Therefore, long-range chromosomal contacts may explain how SNPs within distal regulatory elements convey the risk to certain diseases. Furthermore, loop formation depends on an open chromatin conformation around the interacting genomic elements. Thus, mutations in structural chromatin proteins (CTCF, cohesins) and chromatin modifying enzymes (Setdb1, MLL1) may alter chromatin condensation around a locus and abolish or promote loop formation, which in turn induces transcriptional changes that may result in cognitive dysfunction (Dekker, 2003; Mitchell et al., 2014; Rajarajan et al., 2018b).

Chromatin looping has only recently been discovered as a factor in neuronal dysfunction and its role in many diseases remains largely unexplored. For example, a 2019 large scale study surveyed >40,000 GWAS-identified genetic variants and their associated eQTL genes, among them Parkinson-associated SNPs that appeared to influence expression of distant genes (Jung et al., 2019). However, so far there are no publications on the 3D genome of Parkinson’s disease.

**FIGURE 1:**
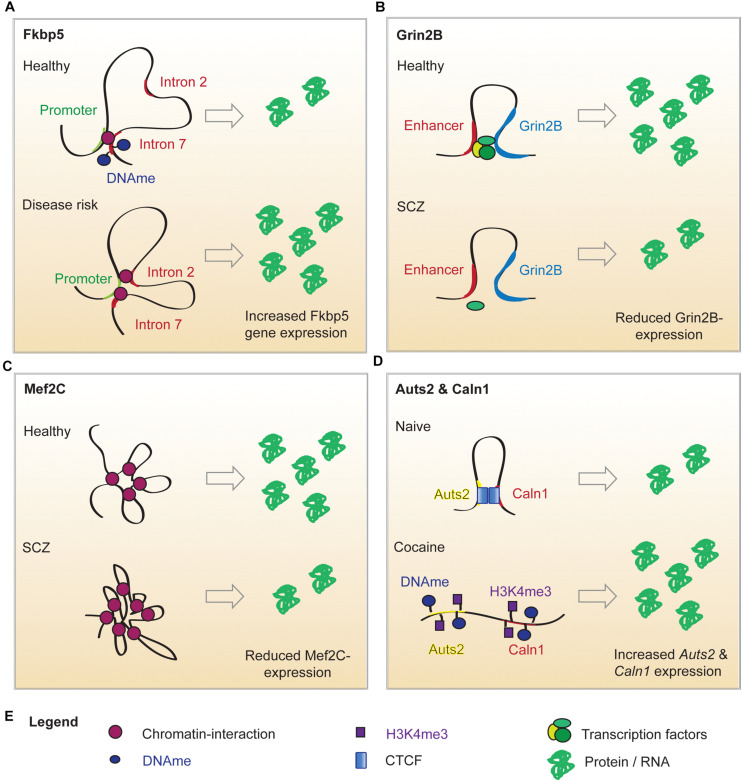
Putative mechanisms of altered chromatin looping in mental illness. **(A)** Under normal conditions, the Fkbp5 promoter forms a chromatin loop with intron 7 of the Fkbp5 gene. When a risk allele is present, a second chromatin interaction between the promoter and intron 2 of the Fkbp5 gene forms. In consequence, *Fkbp5* expression is increased and results in an altered stress response. **(B)** In healthy brains, *Grin2b* expression is upregulated by an enhancer-promoter loop. An SCZ-related SNP in the enhancer sequence decreases its’ ability to bind proteins. This has been hypothesized to impair enhancer binding to TFs and sharing them with the Grin2b locus. This could explain the lowered *Grin2b* expression in a subset of SCZ patients. **(C)** *Mef2c* expression is regulated by interactions with twelve long distance partners. A SCZ-related SNP in a distant enhancer leads to extensive loop interaction with the locus and ultimately results in lower *Mef2c* expression. **(D)** Cocaine administration alters the local chromatin landscape surrounding Auts1 and Caln2, which abolishes the looping interaction. This is associated with increased DNA methylation (DNAme), reduced CTCF binding and elevated levels of the permissive chromatin mark H3K4me3. This is associated with an elevated expression of the two genes.

Genome-wide association studies (GWAS) also revealed that SNPs implicated in major depressive disorder (MDD) are mostly found in non-coding sequences posing the question by which gene regulatory mechanisms they may influence transcription (Wray et al., 2018). A particularly promising target may be the FK506 binding protein 51 (FKBP5) locus. *Fkbp5* encodes FK506 binding protein 51, which is a glucocorticoid receptor (GR) co-chaperone (Klengel and Binder, 2015). *Fkbp5* transcription is induced by GR binding to GR response elements within Fkbp5 introns. The FKBP5 protein product in turn binds to GRs and prevents their activation by glucocorticoids, creating an “ultra short” negative feedback (Klengel and Binder, 2015). Therefore, disturbance of *Fkbp5* expression alters stress response and resilience (Klengel and Binder, 2015). 3C experiments in human cells revealed that a SNP in the GR response element within intron 2 of the Fkbp5 gene can induce an aberrant long-range interaction of this GR response element with the TSS of Fkbp5, with the result of increased Fkbp5 expression (Klengel et al., 2013) (Figure 1A). This is accompanied by demethylation of the GR response element in intron 7 of the Fkbp5 gene, which further enhances transcriptional activity around the Fkbp5 locus (Klengel et al., 2013). Individuals carrying the risk allele exhibit elevated cortisol levels in response to environmental stressors for prolonged time periods. They also show an altered activity in brain regions involved in startling responses, which is associated with a higher risk to develop MDD or post-traumatic stress disorder following childhood trauma (Klengel and Binder, 2015). This is the first example of aberrant genomic 3D interactions impacting on susceptibility to depressive-like behaviors that may underlie a plethora of stress-associated conditions such as SCZ, bipolar disorder, aggression, psychosis and suicide (Daskalakis and Binder, 2015).

The neuronal chromatin interactomes involved in MDD and Parkinson’s disease, as well as in many other mental illnesses, are only beginning to be understood. However, 3D chromatin dynamics are an emerging field that has already uncovered novel gene regulatory mechanisms that when disturbed impede neuronal function and it is likely advance our understanding of brain health and disease. Below, emerging evidence for chromatin architecture in Alzheimer’s disease (AD) is presented, and findings on the role of long-range chromatin contacts in SCZ and addiction are discussed.

## Alzheimer’s Disease

Worldwide, more than 30 million people suffer from AD, the most common type of dementia^1^. An estimated AD heritability of about 65% (Kikuchi et al., 2019) gives hope that the underlying genetic mechanisms could hold the key to a cure as well. Bioinformatics analysis of public data sets revealed that >95% of AD-associated SNPs lie outside of coding sequences. Almost a third of them is located in enhancer sequences of expression quantitative trait loci (eQTLs) involved in amyloid β clearance, synaptic transmission and immune response (Kikuchi et al., 2019). These findings were strengthened by HiC experiments that showed the colocalization of SNP-bearing enhancers with their associated eQTL genes in the same TADs (Kikuchi et al., 2019) (Table 1). This observation was especially pronounced for 19 enhancers carrying SNPs within specific TF binding motifs (Kikuchi et al., 2019). Accordingly, the authors of this study generated a list of candidate SNPs that potentially promote AD progression through specific long-range interactions. Furthermore, they identified a SNP in a region that forms CTCF-bound loops with Paired Immunoglobin Like Type 2 Receptor Alpha and Beta (PILRA, PILRB) and Stromal antigen 3 opposite strand (GATS) gene loci, which have been shown to be dysregulated in various brain structures affected by AD (Kikuchi et al., 2019). Another contact locus was the TF Sterol Regulatory Element Binding Transcription Factor 2 (SREBF2), which regulates several genes that are strongly implicated in AD (Kikuchi et al., 2019). Thus, there is indirect evidence that 3D chromatin dynamics are relevant to AD. These findings exemplify the need to take protein interaction networks into account, when gene expression patterns and long-range interactions are concurrently analyzed (Kikuchi et al., 2019).

**TABLE 1:** Studies on chromatin looping in patients and rodent models.

Disorder	Human tissue	Animal model	Cell line
AD	Association of SNPs and eQTLs in same TADs (Kikuchi et al., 2019)		SNP rs1476679 rs7364180 forms chromatin loops with AD-associated eQTL genes (Kikuchi et al., 2019)
SCZ	SCZ-associated H3K4me3 peak forms loop interactions with BTNL2 and HLA-DRB5 genes (Dekker and Mirny, 2016) Low input HiC of midbrain dopaminergic neurons from healthy subjects: intra- and trans-chromosomal contacts from between SCZ and BMI risk loci and are enriched for specific eQTLs (Watson and Tsai, 2017) Presence of SNP rs117578877 in one of the Grin2b-interacting sequences was associated with reduced Grin2b mRNA and increased schizotypy (Espeso-Gil et al., 2020)	Gad1 promoter interacts with multiple SCZ-associated loci (Dekker, 2003) 3C in PFC neurons of transgenic mice either overexpressing Setdb1 or KO of MLL1: reduced loop formation, Grin2b expression and working memory (Espeso-Gil et al., 2020)	hIPSCs were differentiated into neuronal progenitor cells, neurons and glia. HiC analysis found long range chromosomal contacts involving SCZ risk loci (Mitchell et al., 2016) 3C on cultured neurons from SCZ patients show that Mef2c TSS forms 12 loops + SCZ neurons had ↑loop formation between Mef2c TSS and sequence containing SNP rs16867576 (Rajarajan et al., 2018a) Grin2b interaction partner carrying SNP rs117578877 has decreased ability to bind proteins (Espeso-Gil et al., 2020)
Addiction		Cocaine-induced opening of chromatin loop between Auts2 and Caln1 genes (Rajarajan et al., 2018b)	CRISPR-epigenome editing: DNA methylation altered across cocaine-regulated loop (Rajarajan et al., 2018b)
Stress-related disorders	Contact between SNP rs1360780 and Fkbp5 intron 2 is associated with increased risk to develop posttraumatic stress disorder (Sartorelli and Lauberth, 2020)		Presence of SNP rs1360780 disturbed loop formation between Fkbp5 TSS and intron 2 in lymphoblastoid cell lines (Sartorelli and Lauberth, 2020)

*AD, Alzheimer’s Disease; eQTL, expression quantitative trait loci; 3C, chromatin conformation capture; HiC, high throughput chromatin conformation capture; SCZ, schizophrenia; Grin2b, glutamate receptor ionotropic, NMDA 2B; TSS, transcription start site; SNP, single nucleotide polymorphism; DAM-ID, DNA adenine methyltransferase identification; Gad1, glutamate decarboxylase 1; Setdb1, histone-lysine N-methyltransferase SETDB1; KO, knock out; MLL1, Histone-lysine N-methyltransferase 2A; hIPSCs, human induced pluripotent stem cells; Mef2c, myocyte-specific enhancer factor 2C; Auts2, autism candidate 2; Caln1, Calcium-binding protein 8; 4C, chromatin conformation capture on chip; Fkbp5, peptidyl-prolyl cis-trans isomerase FKBP5; WT, wild type.*

Another layer of complexity to higher chromatin structures in AD is added, when chromatin marks around enhancers are investigated. Originally, RNA sequencing in AD postmortem tissue revealed an overexpression of many enzymes involved in chromatin modifications in the lateral temporal lobe (Nativio et al., 2020). This finding prompted Berger’s team to use LC-MS/MS for the screening of histone marks in postmortem tissues to define histone signatures corresponding to normal and pathological aging (Nativio et al., 2020). The most pronounced difference between the healthy and AD cohort was the enrichment of H3K9 and H3K27 acetylation (Nativio et al., 2020). Further analyses showed that not only global levels but also the deposition pattern of chromatin marks across the genome (H3K9ac, H3K27ac, H3K4me3, cytosine methylation) was altered in AD samples (Nativio et al., 2020). A reduction in histone acetylation correlated most frequently with the loss of H3K4me3 and DNA methylation, whereas disease-specific gains of H3K9ac and H3K27ac occurred independently of the other analyzed marks (Nativio et al., 2020). Notably, histone hyperacetylation was not associated with TSSs, which suggests that it indirectly affects gene expression (Nativio et al., 2020). The sites of disease specific gains of H3K9ac and H3K27ac were analyzed for protein binding sequences, which revealed an enrichment for the Nuclear respiratory factor 1 (NRF1) and CTCF motifs (Nativio et al., 2020). NRF1 has been shown to act as enhancer-bound TF that reaches its target through looping (Bharadwaj et al., 2014), while CTCF has been implicated in loop formation and stabilization (Nativio et al., 2020). Thus, there is a potential for AD-specific alterations to contribute to aberrant chromosomal loops (Nativio et al., 2020). As the disease-specific gains in H3K9 and H3K27 acetylation overlapped with GWAS-identified risk SNPs, the consequences on chromatin architecture might be heritable as well (Nativio et al., 2020). The physiological relevance of the AD-associated acetylation peaks was elegantly proven in a fly model of AD, where transfection with H3K9ac/H3K27ac mimics enhanced amyloid-β-42 associated neurodegeneration (Nativio et al., 2020). This warrants studies to investigate the overlay between AD-specific H3K9/27ac peaks and HiC derived chromosome contact maps.

## Schizophrenia

Despite its’ high incidence and considerable research efforts, SCZ remains poorly understood and this is reflected by the lack of an effective treatment strategy (Rajarajan et al., 2018b). Part of the problem is the vast number of genetic variants and affected protein expression networks that might elicit similar clinical symptoms by distinct mechanisms (Rajarajan et al., 2018b; Gusev et al., 2019). The SNPs associated with SCZ risk are predominantly found within enhancer sequences. Hence, the group of Akbarian and colleagues has launched large scale investigations of SCZ-related changes in chromatin architecture. In 2014, the research group identified brain-specific promoter-enhancer loops that regulate the expression of Grin2b and found that SCZ-SNPs in the enhancer reduced its ability to bind proteins (Bharadwaj et al., 2014) (Figure 1B). Therefore, the SNP carriers might have an enhancer lacking the ability to bind relevant TFs and share them with the Grin2b locus (Bharadwaj et al., 2014). This is in line with the observation that lower levels of Grin2b mRNA were found in postmortem PFC samples of SCZ patients carrying the risk allele (Bharadwaj et al., 2014). In a cohort of healthy, young men (Greek Learning on Genetics of Schizophrenia Spectrum cohort), presence of the SNP correlated with SCZ-associated personality traits and working memory deficits (Bharadwaj et al., 2014). The causality of these observations was proven in a mouse model: overexpression of SET Domain Bifurcated Histone Lysine Methyltransferase 1(Setdb1), a H3K9 methyltransferase (inactivating mark) and known regulator of Grin2b expression, caused local heterochromatinization around the Grin2b locus, which abolished its long-range interactions and Grin2b expression and reduced working memory in mice (Bharadwaj et al., 2014). Interestingly, knockout of a H3K4 demethylase in a second mouse cohort (H3K4me is an activating mark) could recapitulate these findings (Bharadwaj et al., 2014). This data is limited, as overexpression and knockout of a histone modifying enzyme will have genome wide effects, but it offers a mechanistic explanation for Setdb1’s effect on Grin2b expression and implies that genetic and epigenetic variation around regulatory elements and gene loci can contribute to aberrant loop-interactions and hence gene expression, which alters cognitive performance (Bharadwaj et al., 2014). The connection between neuronal development, chromatin marks and looping was further strengthened by a study focused on Myocyte Enhancer Factor 2C (Mef2c), a TF whose binding motifs are enriched in promoters and enhancers of neuronal genes (Mitchell et al., 2018). Analysis of the BrainCloud dataset containing RNAseq data generated from postmortem brain tissues of neurotypical subjects across the human lifespan, showed that Mef2c undergoes substantial expression changes during normal development (Mitchell et al., 2018). Together with the observation that H3K4 methylation appears to be enriched in *cis*-regulatory elements and is dysregulated in immature neurons of SCZ patients, this prompted the authors to determine the H3K4me3 pattern in neuronal nuclei derived from postmortem PFC tissues (Mitchell et al., 2018). They discovered almost 1,500 genomic sites with differential H3K4 methylation in SCZ compared to the control group (Mitchell et al., 2018). Mapping these differentially methylated regions exhibited no correlation with promoters or TSSs, but indicated that active enhancers were overrepresented at peaks of H3K4 hypermethylation (Mitchell et al., 2018). The enhancers in question were enriched for Mef2c binding sequences. Thus, it was hypothesized that the increase in open chromatin marks around the enhancers could be a compensatory mechanism for diminished Mef2c availability (Mitchell et al., 2018). Indeed, a siRNA-mediated knockdown of Mef2c caused a significant increase in H3K4 methylation at Mef2c targets *in vitro* (Mitchell et al., 2018). These findings corroborate the notion that Mef2c activity is lacking in SCZ PFC and thus, prompted the authors to evaluate the effect of Mef2c overexpression on cognitive performance (Mitchell et al., 2018). Supraphysiological levels of Mef2c in PFC neurons was verified in mice infected with genetically altered viruses. Transcriptional activity on those genes was increased, which contained Mef2c motifs and exhibited H3K4 hypermethylation in the SCZ PFC (Mitchell et al., 2018). This transcriptomic program significantly improved short and long term memory in a pharmacologically induced SCZ mouse model and might be associated with modified spine morphology (Mitchell et al., 2018). In a last set of experiments the authors took a closer look at the regulation of Mef2c by long range chromosomal interactions and found that the Mef2c locus is involved in twelve loops, one of them connecting Mef2c with a regulatory sequence carrying a SCZ SNP (Mitchell et al., 2018). The SNP significantly increased loop formation with Mef2c in neuronal cultures from SCZ patients (Figure 1C), however, as this was not recapitulated *in vivo* or in postmortem PFC, the functional relevance of this finding remains to be proven (Mitchell et al., 2018). The present study identified Mef2c expression as a intervention point in the context of SCZ, which is currently a much more feasible therapeutic approach compared with epigenome or genome editing approaches (Mitchell et al., 2018). Additionally, Mitchell et al. present evidence for a role of H3K4 methylation in specific SCZ-related genes, which is in agreement with a more recent study demonstrating that regions with differentially methylated H3K4 do not exhibit general overlap with SCZ-associated SNPs (Mitchell et al., 2018; Gusev et al., 2019). These findings suggest that a specific SCZ-related SNP can disrupt loop formation with the Mef2c promoter, which results in decreased Mef2c expression observed in postmortem PFC of SCZ patients. The reduced availability of this TF is compensated for by chromatin decondensation around its binding motifs – marked by H3K4 hypermethylation. The Mef2c binding motifs affected by H3K4 hypermethylation are enriched for enhancers, which suggests that altered Mef2c levels/activity can affect the activity of enhancers and may promote a SCZ-related transcriptomic program, which can be reversed by Mef2c overexpression.

Schizophrenia (SCZ) is thought to be promoted by the disruption of neurodevelopmental processes long before the first functional symptoms arise. This prompts research into aberrant chromatin looping during brain development (Mitchell et al., 2016). Schizophrenia might be connected to the large scale loop pruning event observed in neuronal differentiation from neural progenitor cells (Rajarajan et al., 2018a). The neuron-specific remodeling of long range chromatin interactions is believed to foster a transcriptional program pertinent to neuronal function (Rajarajan et al., 2018a). Therefore, the authors hypothesize that incomplete or faulty chromatin remodeling could contribute to the development of SCZ (Rajarajan et al., 2018a). The same study found that neurons are the cell type with the highest number of loops in known SCZ risk loci (Rajarajan et al., 2018a). In order to connect a disruption of brain development with adult-onset psychiatric disorders, disease-associated mechanisms must be longitudinally assessed (Mitchell et al., 2016). To determine past looping interactions, a pioneering study exploited the sequence-specific binding of a transcription activator-like effector (TALE) protein targeted toward the Glutamate decarboxylase 1 (Gad1) promoter that was fused to a bacterial DNA Adenine methylase (DAM) (Mitchell et al., 2016). DNA methylation was chosen because it is a stable chromatin mark, and DAM was selected as it methylates adenine exclusively within the GATC motif, a modification usually lacking from the vertebrate brain (Mitchell et al., 2016). N-methyl-D-aspartate receptors of mice were transiently inhibited to elicit a SCZ-like phenotype. Then, mice were infected with a herpes simplex-virus to introduce the anti-Gad1-TALE-Dam protein (Mitchell et al., 2016). Brains were harvested two or 22 weeks after viral infection and adenine methylation was assessed across the whole genome of PFC neurons (Mitchell et al., 2016). Thus, adenine methylation at week 22 represented Gad1 locus-associated chromosomal interactions at the time of infection (Mitchell et al., 2016). Additionally, mRNA expression of relevant genes and animal behavior were tested (Mitchell et al., 2016). As expected, reduced Gad1 expression in the PFC of the SCZ mouse-model correlated with altered working memory and anxiety-related behaviors (Akbarian and Huang, 2006; Mitchell et al., 2016). The analysis of adenine methylation showed that two loops involving Gad1 were dysregulated, which is in agreement with 3C results from the present study as well as previous independent 3C libraries in PFC of other SCZ mouse models (Mitchell et al., 2016). This is proof of principle that “DamID” can be used to determine long range chromosomal interactions at the time of transfection. However, this method is only advantageous when past (DamID) and present (3C based technique) loops can be analyzed in concert, i.e., from the same brain region. Otherwise, the present behavior could not be attributed to aberrant chromosome contacts at the previous time point. It should also be noted that adenine methylation might preferentially wash out within genomic regions commonly undergoing DNA strand breakage and damage response as sometimes seen around immediate early genes (Mitchell et al., 2016; Watson and Tsai, 2017). Nevertheless, DamID might prove a valuable tool for future studies of post-traumatic stress disorder and depression as the risk for these disorders depends on past exposures (Mitchell et al., 2016).

The evidence presented above points toward chromatin looping as one crucial mechanism that links SCZ SNPs to altered gene expression in the PFC and, in consequence, altered behavior. In addition, long-range chromosomal contacts might connect genetic variants that are far away on the linear genome yet associated with frequently comorbid phenotypes. The first study to explore this possibility hypothesized that the high incidence of metabolic aberrations and corpulence among SCZ patients could be caused by an association of the metabolic risk loci in 3D (Espeso-Gil et al., 2020). The authors chose to investigate midbrain dopaminergic neurons (MDN) as these are involved in cognition and the regulation of food intake and metabolism (Espeso-Gil et al., 2020). They initially demonstrated that sequence variants related to mood, psychotic disorders and metabolic dysregulation were enriched within enhancers actively involved in the regulation of the MDN-specific transcriptome (Espeso-Gil et al., 2020). This supported the hypothesis that metabolic and psychiatric disorders might both, at least in part, arise in the MDN (Espeso-Gil et al., 2020). A HiC based genomic map of MDN nuclei exhibited physical clustering of SCZ and risk loci for a high body mass index within the same TADs, as well as direct intra- and trans-chromosomal contacts between risk loci (Espeso-Gil et al., 2020). Colocalization of genes implicated in SCZ and metabolic disorders within the same nuclear compartment could point toward their transcriptional coregulation (Espeso-Gil et al., 2020). Further, this might suggest that these genes interact with the same SNP-carrying regions that convey the risk to both, metabolic diseases and SCZ (Espeso-Gil et al., 2020). Moreover, specific loops with enhancers containing the SCZ and metabolic SNPs indicate that they might control each others’ regulatory activity at a target locus (Espeso-Gil et al., 2020). The latter concept could explain the substantial enrichment of brain-specific eQTLs within the loop-bound enhancer regions (Espeso-Gil et al., 2020). In agreement with this, gene ontology terms most strongly associated with the corresponding eQTL genes were adipogenesis, lipid regulation, dopaminergic neurogenesis, neuronal connectivity and reward-addiction pathways (Espeso-Gil et al., 2020).

These findings corroborate the role of chromosomal loops in bringing risk loci associated with different diseases into physical proximity despite their ‘linear separation’, and provide a potentially universal mechanism, by which co-occurrence of certain phenotypes could be promoted. However, as the present study exclusively investigated postmortem specimen of control subjects, it remains to be proven whether the association of risk loci is disturbed in SCZ patients carrying the respective risk alleles. Furthermore, animal studies will be required to pin down the exact molecular mechanisms, by which loops between risk loci contribute to disease-associated transcriptional signatures (Espeso-Gil et al., 2020). Such studies might contribute to a more accurate risk prediction on the development of cognitive and metabolic disorders but also allow for more personalized treatment approaches (Espeso-Gil et al., 2020).

## Addiction

Environmentally induced epigenetic alterations have been proposed to account for the majority of non-heritable components of psychiatric disease risk (Gusev et al., 2019). Intoxication is one such environmental impact. In particular, chronic or repeated drug use has been shown to induce a plethora of changes in chromatin marks, which in turn can lead to persistent modifications of brain regions and may manifest as addiction (Anderson et al., 2019). Drug-induced changes in chromatin architecture were first proven in the nucleus accumbens of rodent models for chronic cocaine use (Engmann et al., 2017). Here, we showed that chromatin looping of the Autism candidate 2 (Auts2) locus is dynamically altered by cocaine. Auts2 is the gene with most cocaine-induced chromatin modifications (Feng et al., 2014) and co-incidently the most dynamically evolved gene between Homo sapiens and Neanderthal man (Green et al., 2010).

4C revealed a long range intrachromosomal interaction between Auts2 and the Calneuron 1 gene (Caln1) ∼1.5Mb apart, which was disrupted by cocaine consumption in rats and mice (Engmann et al., 2017) (Figure 1D). This cocaine-induced release of the Auts2-Caln1 loop was accompanied by increased Auts2 and Caln1 mRNA expression and altered chromatin marks (Engmann et al., 2017). The latter comprised increased levels of DNA cytosine methylation, which might have caused the concurrent loss of CTCF binding (Engmann et al., 2017). Furthermore, the activating mark H3K4 trimethylation was increased on Auts2 and Caln1 loci, which may contribute to the observed transcriptional changes. A CRISPR-dCAS9 based epigenome editing system was used to selectively increase DNA-methylation on either the Caln1 or Auts2 interacting regions in Neuro2A-cells. Remarkably, a selective increase in DNA methylation near the Auts2 promoter increased Caln1 transcription without affecting Auts2 mRNA levels, providing the first causal evidence for chromatin marks on gene expression across a loop (Engmann et al., 2017).

Using transgenic reporter mouse lines we found that transcription of Auts2 and Caln1 was specifically altered in D2-type medium spiny neurons (D2MSN) (Engmann et al., 2017). Additionally, transcriptional changes of both genes were confirmed in nucleus accumbens postmortem tissue of cocaine addicts (Engmann et al., 2017). Viral-mediated overexpression of Auts2 or Caln1 specifically in D2-MSNs promoted cocaine-related behaviors in mice (Engmann et al., 2017). In summary, this study provided a proof of principle that cocaine-induced changes in chromatin looping can alter transcription of gene products that causally contribute to drug-associated behaviors. This finding should encourage future large scale HiC mapping studies to explore whole genome alterations brought about by drug use (Engmann et al., 2017).

## Perspectives

Neurons are particularly sensitive to aberrant gene dosage and the functional consequences greatly vary depending on the developmental state of the brain (Yamasaki et al., 2020). Thus, any mechanism involved in transcriptional regulation can be expected to have deleterious effects if it is disturbed in the brain. Chromatin architecture has been shown to be a crucial player in the control of neuronal gene expression under physiological conditions (Bharadwaj et al., 2014; Espeso-Gil et al., 2020). Furthermore, the alteration of specific long range chromosomal interactions had effects on gene expression and behavioral phenotypes in a cocaine addiction model (Engmann et al., 2017) and SCZ (Bharadwaj et al., 2014; Mitchell et al., 2018).

HiC proof of aberrant intra- and trans-chromosomal contacts in the context of neurodegenerative diseases is currently lacking. However, AD-specific gains in histone acetylation were enriched for CTCF and NRF1 motifs (Nativio et al., 2020) and AD-specific histone hyperacetylation overlapped with some GWAS-identified risk SNPs, which suggests that inappropriate chromosome looping contributes to AD etiology (Nativio et al., 2020). Similarly, there is preliminary evidence implicating chromosome looping in depression and other mood disorders (Zannas and Binder, 2014; Wray et al., 2018). Overall, the role of long-range chromosomal interactions in neurologic disorders remains largely unexplored. In contrast, extensive research in the field of cancer genetics has shed light on mechanisms, by which the chromatin architecture contributes to aberrant gene regulation during cell proliferation and transition that ultimately promote malignant diseases (Kantidze et al., 2020). These efforts have culminated in the development of a curaxin compound (CBL0137) as anticancer drug (Jin et al., 2018; Kantidze et al., 2020).

A fundamental gap in the chromatin field is the regulation of loops by environmental stimuli. Studies on memory formation (Bharadwaj et al., 2014) and cocaine exposure (Engmann et al., 2017) suggest that this is possible and hence, more emphasis should be put on researching this aspect.

New molecular mediators of looping keep being discovered. Recent research identified the chromatin remodeling complex Chd4 as a regulator of chromatin accessibility, cohesin binding and loop formation in the cerebellum (Goodman et al., 2020). Other studies are likely to identify more integrative signaling pathways. Chromatin loop formation can be expected to be an extraordinarily complex process as it incorporates many layers of the epigenome: (1). relative positioning of the interaction partners within the nucleus (2). Accessibility of the interacting sequences, which is governed by DNA methylation and histone modifications (3). Competition with multiple potential partners, which can be other gene loci [as seen for Caln1 and Auts2 (Engmann et al., 2017)] or enhancers [for example observed between the Grin2b locus and distant regulatory sequences (Bharadwaj et al., 2014)]. This is further complicated by the high degree of neuronal plasticity for two reasons. Firstly, the functional consequences of looping interactions might be more subtle than switching a gene on or off. Certain epigenetic signatures do not alter gene expression but prime for transcription. Thus, it is likely that formation of a chromatin loop can only affect gene expression under a certain set of conditions, i.e., presence of other epigenetic marks or binding of specific protein factors. Secondly, the role of chromatin conformation in the neurodevelopmental origin of neurologic diseases is difficult to study because tracking of the 3D genome over time is currently impossible. Alteration of chromosome contacts during brain maturation could explain how disease-associated SNPs may contribute to an abnormal phenotype, even though they are reticent in adulthood. The DamID technique utilized by Mitchell et al., comprises a first step toward a longitudinal assessment of long-range chromosomal contacts that could test whether SNP-bearing candidate sequences change their contact status or partners over the course of differentiation (Mitchell et al., 2016). The exact interactions between regulatory elements, TFs and chromatin modifiers acting at a single locus can be expected to be unique and thus are likely to contribute to disease heterogeneity. Nevertheless, they may be uncovered by the curation of whole genome maps that contain information on histone marks, DNA methylation, TF occupancy, long range chromosomal contacts, chromatin accessibility, and genomic sequence for all brain regions under physiological and disease conditions. This endeavor may be realized through global scientific corporation and the rapidly expanding tool box to study the (epi-)genome and ultimately allow us to precisely diagnose patients and offer them adequate and personalized treatment strategies. Considering the dynamic nature of the brain and the epigenome, it might even contribute to custom-tailored treatments for certain disorders in the future.

## Author Contributions

MB wrote the manuscript. OE provided the idea for the manuscript, edited the manuscript, and designed the figure. Both authors contributed to the article and approved the submitted version.

## Conflict of Interest

The authors declare that the research was conducted in the absence of any commercial or financial relationships that could be construed as a potential conflict of interest.

## Publisher’s Note

All claims expressed in this article are solely those of the authors and do not necessarily represent those of their affiliated organizations, or those of the publisher, the editors and the reviewers. Any product that may be evaluated in this article, or claim that may be made by its manufacturer, is not guaranteed or endorsed by the publisher.

## Footnotes

1
https://www.alzint.org/about/dementia-facts-figures/dementia-statistics/
